# Supplementary material for: On the estimation of the fill rate for the continuous (s, S) inventory system for the lost sales context
Source: PLoS One. 2022 Feb 17;17(2):e0263655. doi: 10.1371/journal.pone.0263655 (PMC8853522; doi:10.1371/journal.pone.0263655)
Supplement: S1 Appendix — (DOCX) [file pone.0263655.s002.docx]

**S1 Appendix. *Calculation of the transition matrixes* and**

The components of the probability transition matrix of the states from the beginning of the cycle until the stock reaches the ROP are:

(1)

When the ROP is reached, the on-hand stock balance may be expressed as $j=\left[i-D_{\tau}\right]^{+}$, which leads to the identification of two possible situations:

1. when there is a stockout at the very moment the ROP is reached and therefore

$P\left(D_{\tau}\geq i\right)=1-P\left(D_{\tau}<i\right)=1-H\left(i-1\right)$,

1. in the opposite case, when on-hand stock remains on the shelves when the ROP is reached, which implies that $P\left(D_{\tau}=i-j\right)=h\left(i-j\right)$.

Therefore:

$P\left(z_{\tau}=j/z_{0}=i\right)=\left\{\begin{matrix} 1-H\left(i-1/i\right) j=0 \\ h\left(i-j/i\right) j>0 \end{matrix}\right.$ (2)

Analogously:

(3)

In this case, the on-hand stock balance at the beginning of the cycle, just upon order delivery and taking into account the lost sales context, may be expressed as $j=\left[j-D_{L}\right]^{+}+S-j$, which leads to the identification of two possible situations:

1. when demand during the lead time is equal to or higher than the on-hand stock when the ROP is reached and therefore the on-hand stock is depleted during *L*, so$P\left(D_{L}>j\right)=1-F_{L}\left(j-1\right)$; and
2. when demand during the lead time is lower than the on-hand stock when the ROP is reached, therefore $P\left(D_{L}=S-k\right)=f_{L}\left(S-k\right)$.

Therefore:

$P\left(z_{\tau+L}=k/z_{\tau}=j\right)=\left\{\begin{matrix} 1-F_{L}\left(j-1\right) k-S+j=0 \\ f_{L}\left(S-k\right) k-S+j>0 \end{matrix}\right.$ (4)
