# Supplementary material for: On the estimation of the fill rate for the continuous (s, S) inventory system for the lost sales context
Source: PLoS One. 2022 Feb 17;17(2):e0263655. doi: 10.1371/journal.pone.0263655 (PMC8853522; doi:10.1371/journal.pone.0263655)
Supplement: S2 Appendix — (DOCX) [file pone.0263655.s003.docx]

**S2 Appendix. Derivation of the demand distribution from the beginning of the cycle until ROP.**

Let $\vartheta$ be the quantity of demand needed to reach the ROP, hence:

$h\left(D_{\tau}/z_{0}\right)=P\left(D_{\tau}=\vartheta/D_{\tau}\geq z_{0}-s\right)=\frac{P\left(D_{\tau}=\vartheta\cap D_{\tau}\geq z_{0}-s\right)}{P\left(D_{\tau}\geq z_{0}-s\right)}$ (1)

Let $T$be the time required for the demand to equal $\vartheta$ then the numerator of the expression (1) must be expanded taking into account the probability that $P\left(T=\tau\right)$in the following way:

$P\left(D_{\tau}=\vartheta\cap D_{\tau}\geq z_{0}-s\right)=\sum_{\tau=1}^{\infty} P\left(D_{\tau}=\vartheta\cap D_{\tau}\geq z_{0}-s/T=\tau\right)\cdot P\left(\tau\right)$ (2)

Reaching the ROP in $\tau$ units of time implies that the accumulated demand over those $\tau$ units of time is greater than or equal to the on-hand stock at the beginning of the cycle minus the on-hand stock at the Standard moment the ROP is reached; i.e. $P\left(T\leq\tau\right)=P\left(D_{\tau}\geq z_{0}-s\right)=1-F_{\tau}\left(z_{0}-s-1\right)$. Given that $P\left(T=\tau\right)=P\left(T\leq\tau\right)-P\left(T\leq\tau-1\right)$:

$P\left(\tau\right)=\left\{\begin{matrix} 1-F_{\tau}\left(z_{0}-s-1\right) \tau=1 \\ F_{\tau-1}\left(z_{0}-s-1\right)-F_{\tau}\left(z_{0}-s-1\right) \tau>1 \end{matrix}\right.$ (3)

On the basis of the value of $\tau$situations are identified:

1. $\tau=1$, which implies that the demand over a unit of time has to be equal to $\vartheta$, and therefore $P\left(D_{\tau}=\vartheta\cap D_{\tau}\geq z_{0}-s/\tau=1\right)=f\left(\vartheta\right)$;
2. $\tau>1$, which implies that two situations arise simultaneously: (1) the demand accumulated over $\tau-1$ unit of times is insufficient for the ROP to be reached, i.e. $D_{\tau-1}=\psi$ with $\psi<\vartheta$; and (2) at the moment that elapses when $\tau-1$ becomes $\tau$, the demand has to be sufficient for the ROP to be reached, i.e. $d_{1}=\vartheta-\psi$. Therefore:

$$\begin{matrix} P\left(D_{\tau}=\vartheta\cap D_{\tau}\geq z_{0}-s/\tau>1\right)= \\ =\sum_{\psi=0}^{z_{0}-s-1} P\left(D_{\tau-1}=\psi\cap d_{1}=\vartheta-\psi\right)=\sum_{\psi=0}^{z_{0}-s-1} f_{\tau-1}\left(\psi\right)\cdot f\left(\vartheta-\psi\right) \end{matrix}$$

Thus:

$\begin{matrix} P\left(D_{\tau}=\vartheta\cap D_{\tau}\geq z_{0}-s\right)=f\left(\vartheta\right)\cdot\left(1-F_{\tau}\left(z_{0}-s-1\right)\right)+ \\ \sum_{\tau=2}^{\infty} \left[\left(F_{\tau-1}\left(z_{0}-s-1\right)-F_{\tau}\left(z_{0}-s-1\right)\right)\cdot\sum_{\psi=0}^{z_{0}-s-1} f_{\tau-1}\left(\psi\right)\cdot f\left(\vartheta-\psi\right)\right] \end{matrix}$ (4)

Finally:

$\begin{matrix} P\left(D_{\tau}\geq z_{0}-s\right)=\sum_{\vartheta=z_{0}-s}^{\infty} \left(D_{\tau}=\vartheta/D_{\tau}\geq z_{0}-s\right)= \\ =\sum_{\vartheta=z_{0}-s}^{\infty} \left[\begin{matrix} f\left(\vartheta\right)\cdot\left(1-F_{\tau}\left(z_{0}-s-1\right)\right)+ \\ \sum_{\tau=2}^{\infty} \left[\left(F_{\tau-1}\left(z_{0}-s-1\right)-F_{\tau}\left(z_{0}-s-1\right)\right)\cdot\sum_{\psi=0}^{z_{0}-s-1} f_{\tau-1}\left(\psi\right)\cdot f\left(\vartheta-\psi\right)\right] \end{matrix}\right] \end{matrix}$ (5)

And therefore:

$h\left(D_{\tau}/z_{0}\right)=\frac{\begin{matrix} f\left(\vartheta\right)\cdot\left(1-F_{\tau}\left(z_{0}-s-1\right)\right)+ \\ \sum_{\tau=2}^{\infty} \left[\left(F_{\tau-1}\left(z_{0}-s-1\right)-F_{\tau}\left(z_{0}-s-1\right)\right)\cdot\sum_{\psi=0}^{z_{0}-s-1} f_{\tau-1}\left(\psi\right)\cdot f\left(\vartheta-\psi\right)\right] \end{matrix}}{\sum_{\vartheta=z_{0}-s}^{\infty} \left[\begin{matrix} f\left(\vartheta\right)\cdot\left(1-F_{\tau}\left(z_{0}-s-1\right)\right)+ \\ \sum_{\tau=2}^{\infty} \left[\left(F_{\tau-1}\left(z_{0}-s-1\right)-F_{\tau}\left(z_{0}-s-1\right)\right)\cdot\sum_{\psi=0}^{z_{0}-s-1} f_{\tau-1}\left(\psi\right)\cdot f\left(\vartheta-\psi\right)\right] \end{matrix}\right]}$ (6)
